# Optimization of a Microplate Assay for Generating *Listeria Monocytogenes*, *E. Coli* O157:H7, and *Salmonella* Biofilms and Enzymatic Recovery for Enumeration

**DOI:** 10.3390/foods8110541

**Published:** 2019-11-02

**Authors:** Manish Aryal, Preetty Pranatharthiharan, Peter M. Muriana

**Affiliations:** 1 Robert M. Kerr Food & Agricultural Products Center, Oklahoma State University, Stillwater, OK 74078, USA; aryalm3@gmail.com (M.A.); preettyedox@gmail.com (P.P.); 2 Department of Animal and Food Sciences, Oklahoma State University, Stillwater, OK 74078, USA

**Keywords:** biofilm, carboxyfluorescein diacetate, *Listeria monocytogenes*, *Salmonella*, *E. coli* O157:H7, microplate assay, enzymes

## Abstract

Biofilms enable the persistence of pathogens in food processing environments. Sanitizing agents are needed that are effective against pathogens entrapped in biofilms that are more difficult to inactivate than planktonic cells that are displaced and found on equipment surfaces. We examined conditions to develop, analyze, and enumerate the enhanced biofilms of three different foodborne pathogens assisted by fluorescence adherence assay and enzymatic detachment. We compared three different isomeric forms of fluorescent substrates that are readily taken up by bacterial cells based on carboxy-fluorescein diacetate (5-CFDA, 5,6-CFDA, 5,6-CFDA, SE). Biofilm-forming strains of *Escherichia coli* O157:H7 F4546 and *Salmonella* Montevideo FSIS 051 were identified using a microplate fluorescence assay defined previously for *L. monocytogenes*. Adherence levels were determined by differences in relative fluorescence units (RFU) as well as recovered bacterial cells. Multiple hydrolytic enzymes were examined for each representative pathogen for the most suitable enzyme for detachment and enumeration to confirm adherence data obtained by fluorescence assay. Cultures were grown overnight in microplates, incubated, washed and replenished with fresh sterile growth medium; this cycle was repeated for seven consecutive days to enrich for robust biofilms. Treatments were performed in triplicate and compared by one-way analysis of variance (ANOVA) to determine significant differences (*p* < 0.05).

## 1. Introduction

The development of a biofilm usually involves a cellular adherence event that develops into irreversible attachment followed by development of a 3-dimensional biofilm that progresses into a mature and intricate biofilm [1,2]. During this progression, individual cells or portions of biofilm may slough off that are distributed elsewhere [3]. The initial or reversible attachment of planktonic cells to surfaces involves hydrophilic/hydrophobic interactions whereas the subsequent irreversible attachment may be due to the development of stronger covalent bonds as demonstrated by bacterial lectins and fimbriae [4,5,6]. Attachment is affected by the physiochemical properties of the surface, hydrodynamics, bacterial properties, and may also involve quorum sensing [7]. After attachment, micro-colonies are rapidly formed and the secretion of extracellular polysaccharides (EPS) start to develop, becoming the ‘glue’ of the biofilm architecture. As biofilms mature, higher densities of EPS, channels, and pores results in the positioning of bacteria away from the substrate surface and facilitates the release of planktonic cells or sloughing off as displaced biofilm particles [8]. This can occur due to environmental shear forces, fluid dynamics, or abrasion [7]. Biofilms are generally problematic wherever they are found and may cause biofouling on the bottom of boats [9], in plumbing systems [10], on medical devices (intravenous catheters) and dental surfaces (plaque) [11,12]. Biofilms may also be involved in adverse health consequences when found on equipment surfaces in food manufacturing facilities [13,14,15].

Methods used to quantify bacterial adherence on surfaces have ranged from crystal violet staining (microscopic view) or absorbance readings [16,17] to sophisticated modern methods using 3-dimensional non-destructive analysis [18]. An in-situ fluorescence assay to assess the relative degree of attached bacteria has been implemented based on flow cytometry technique whereby individual fluorescing cells are quantified as they pass a laser beam. This procedure was used to screen adherence properties of numerous strains of *Listeria monocytogenes* [19]. Many strategies have been developed to disperse biofilms, as they pose a threat in food industries, dairy plants, prosthetic devices, human health (plaque) and many other areas [7,20,21]. The use of hydrolytic enzymes has been used in sanitation regimens to detach or disintegrate biofilms found in food processing plants [19,22]. Because of the repeated occurrence of in-plant contamination problems with *L. monocytogenes*, *E. coli* O157:H7, and *Salmonella* serovars, we examined improvements to a method to create robust biofilms with hardy biofilm formers to facilitate biofilm research. The current work examined multiple strains of three pathogens, fluorescent substrates, and hydrolytic enzymes to recover and enumerate these bacteria from biofilms.

## 2. Materials and Methods

### 2.1. Bacterial Strains and Growth Conditions

A variety of *E. coli* O157:H7, *L. monocytogenes*, and *Salmonella* serovars and strains from our culture collection were screened by a microplate fluorescence adherence assay to confirm or identify high level adherence. Strongly-adherent strains were then further used to optimize biofilm formation and enzyme detachment (enumeration recovery) assays such that they could be used for the evaluation of sanitizers in subsequent studies. Cultures were stored frozen by centrifuging 9 mL of overnight culture and resuspending cell pellets in 2–3 mL of fresh sterile Brain Heart Infusion (BHI, Difco, Becton-Dickenson, Franklin Lakes, NJ, USA) broth containing 10% glycerol. Cell suspensions were then stored in glass vials in an ultra-low freezer (−80 °C). Frozen stocks were thawed and revived by transferring 100 µL into 9 mL of BHI, incubating overnight at 30 °C, and sub-culturing at least twice before use in assays. Microbial enumeration for all the assays was carried out on Tryptic Soy Agar (TSA, Difco) plates, plated in duplicate. Although we screened a variety of strains in our culture collection, the main organisms used in this study were: *L. monocytogenes* 99-38, isolated from ground beef [19], *E. coli* O157:H7 F4546, an outbreak isolate from alfalfa sprouts [23,24], and *Salmonella* Montevideo FSIS 051, an isolate from beef [25].

### 2.2. Microplate Adherence Assay

The fluorescence microplate adherence assay has been used as a qualitative screening measure to identify strongly-adherent bacteria. This method was previously used to identify adherence properties of numerous strains of *L. monocytogenes* isolated from raw and ready-to-eat (RTE) meat processing plants in which *L. monocytogenes* 99-38 was identified as a strongly-adherent strain [19,26,27]. The adherence of *L. monocytogenes* 99-38 (strongly-adherent) and CW 35 (weakly adherent) were confirmed in this study as a control for the 5,6-CFDA fluorescence adherence assay used to screen *E. coli* and *Salmonella* spp. Strains of *Salmonella* spp. obtained from the United States Department of Agriculture, Agricultural Research Service [28], and strains of *E. coli* O157:H7 obtained from S.E. Gilliland [29] were screened to identify strongly-adherent strains that could serve as good biofilm formers.

Various parameters were then tested on biofilms of select pathogens grown in microplates such as type of fluorescent dye, number of washes, age of biofilms, and enzymatic release of attached cells for microbial enumeration before settling on a standardized assay prior to use in testing sanitizers against biofilms produced by these organisms.

#### 2.2.1. Fluorescence Substrate for Fluorescence Microplate Assay

The single-isomer substrate 5-carboxyfluorescein diacetate (5-CFDA) and mixed-isomer substrates 5,6-carboxyfluorescein diacetate (5,6-CFDA) and 5,6-carboxyfluorescein diacetate, succinimidyl ester (5,6-CFDA, SE; Molecular Probes/Invitrogen, Carlsbad, CA, United States) were compared for the ability to produce fluorescence signals in a microplate biofilm assay and hence to determine which one was a more suitable substrate for our application. The fluorescent dyes were dissolved in dimethyl sulfoxide (DMSO, Sigma-Aldrich, St. Louis, MO, USA) to get 2% (*w/v*) stock solutions. Working solutions were prepared thereafter by allocating 10 µL of the stock solutions to 1 mL of Tris buffer (0.05 M, pH 7.4). The best performing fluorophore above was incubated with serial dilutions of planktonic cells of *L. monocytogenes* 99-38 to determine if the fluorophore would be overwhelmed by high cell levels that are likely to be present in extended biofilms. Fluorescence emission was read in a Tecan GENios plate reader (Phenix Research Products, Hayward, CA, USA) using a fixed signal gain of 75% with excitation at 485 nm and detection at 535 nm and results were expressed as relative fluorescence units (RFU) [19,30].

#### 2.2.2. Microplates as a Substrate for Attachment and Biofilm Formation

Black, non-treated 96-well flat-bottomed microplates (Cat: 237105, NUNC, Roskilde, Denmark) were used to perform fluorescence assays with adhered bacteria. Black plates prevent “cross-talk” from neighboring wells during fluorescence measurement and fluorescence signals can be read from the top. When fluorescence was not needed, sterile Falcon 96-well clear, non-treated flat-bottomed polystyrene microplates (Cat: 351172, Corning Inc., Corning, NY, USA) were used to grow microbial biofilms and perform subsequent washing, enzyme detachment, and enumeration assays.

#### 2.2.3. Microplate Washing

The microplates used for fluorescence or biofilm enumeration assays, were subjected to a wash treatment in a Biotek Elx405 Magna plate washer (Ipswich, Suffolk, UK). This microplate washer was connected to separate liquid supply containers of 10% Clorox disinfecting bleach solution (Clorox Co., Oakland, CA, USA), sterile de-ionized water, 0.05 M Tris buffer (pH 7.4), and additional waste containers. The plate washer has 96 pairs of needles (a longer one for aspiration and a shorter one for dispensing) to draw liquids into, and out of, each of the wells and a shake parameter to shake the plate to re-suspend settled cells or release loosely adhered cells before washing. Maintenance cycles were performed to sanitize the plate washer needles and tubing by washing with 10% Clorox bleach solution (2 times), followed by sterile de-ionized water (3 times), and sterile Tris buffer (2 times) before and after use with biofilm-containing plates.

In order to determine how many washes were sufficient to remove loosely adhered cells from microplates prior to enzymatic treatment, we set up a series of plates that would be washed 1–4 times with 0.05 M Tris buffer (pH 7.4) using the ‘shake’ option in the Elx405 plate washer during each wash. After each wash series, buffer was added manually to microplates, shaken for 10 s, and then recovered and plated for enumeration of planktonic cell counts.

### 2.3. Enzymatic Detachment of Adhered Cells from Microplates

A variety of enzymes were used that act on different biochemical substrates that may be involved with attachment to surfaces. Previously, we examined similar enzymes for the ability to release *L. monocytogenes* [19]; in this study, we examined a similar set of enzymes for ability to release *L. monocytogenes* 99-38, *E. coli* O157:H7 F4546, and *Salmonella* Montevideo FSIS 051.

#### 2.3.1. Enzymes for Microbial Detachment

Bax protease (DuPont Qualicon, Wilmington, DE, USA) was obtained as a premade solution and used as per manufacturer’s guideline [12.5 µL in 1 mL Tris buffer (0.05 M, pH 7.4)] [19]. The specific protease and concentration/activity is undisclosed as it is a proprietary solution for their PCR kit.

Pronase E (P5147, Sigma-Aldrich, St. Louis, MO, USA) powder from *Streptomyces griseus* (5.3 U/mg) was prepared in Tris buffer (0.05 M, pH 7.4) to obtain a stock solution of 500 U/mL.

Trypsin (T4549, Sigma-Aldrich) from porcine pancreas was obtained in liquid form (1486 U/mL) and was diluted with Tris buffer (0.05 M, pH 7.4) to 500 U/mL.

Papain (5125, EMD Millipore Corp., Billerica, MA, USA) from *Carica papaya* (31,850 U/mg) was prepared in Tris buffer (0.05 M, pH 7.4) to a concentration of 1000 U/mL.

Cellulase (C1184, Sigma-Aldrich, 1.3 U/mg) powder from *Aspergillus niger* (1.3 U/mg) was added to Tris buffer (0.05 M, pH 7.4) to get a desired working stock solution of 500 U/mL.

Lipase (L1754, Sigma-Aldrich) powder from *Candida rugosa* (1170 U/mg) was dissolved in Tris buffer (0.05 M, pH 7.4) to get a working stock solution of 500 U/mL.

Except for the commercially-obtained Bax protease, all of the enzyme solutions were filter-sterilized via 0.22 μm filters and held in the refrigerator or stored frozen (−20 °C) if not used within 1–2 days.

#### 2.3.2. Enzymatic Detachment and Enumeration Assay

The enzymes were evaluated against biofilms of the 3 pathogenic genera to determine which would be the best for recovering bacterial cells from biofilms. Overnight cultures (~9-log CFU/mL) of the three most strongly-adherent pathogenic microbes (one strain from each of the three genera) were diluted to ~4-log CFU/mL in BHI broth. A 200 µL aliquot of each culture was allocated, in triplicate, into Falcon 96-well microplates; each replication of the same organism used a separately-inoculated culture. The microplates were sealed with Parafilm (Fisher Scientific, Waltham, MA, USA) to avoid evaporation and incubated at 30 °C for 24 h. After that, the microplates were washed 3 times with Tris buffer (0.05 M; pH 7.4) in a Biotec Elx405 Magna plate washer as described earlier. A ‘shaking’ option was used to re-suspend settled planktonic cells and loosely held cells. This was followed by the addition of fresh BHI (200 µL) into the wells and an additional incubation for 24 h at 30 °C. The same process of washing with Tris buffer and adding fresh BHI into wells was repeated each day for one week. After 7 days of washing and incubating, the final wash with Tris buffer using the plate washer (with shaking) was performed and 200 µL of different enzymes at the earlier stated concentrations were transferred into the experimental wells. After the addition of enzymes, the microplate was incubated for 1 hour at 37 °C. Finally, to get detached cell counts, the solutions from the wells were further diluted and plated on Tryptic Soy Agar (TSA) plates and incubated at 30 °C for 24–36 h.

### 2.4. Statistical Analysis

Each trial was performed in triplicate replication where each replication was treated as an independent and autonomous experiment using separately inoculated cultures and prepared plating media. All data were presented as the mean of triplicate replications and standard deviation of the mean were represented by error bars. Statistical analysis was done by using one-way analysis of variance (ANOVA) and Holm-Sidak test for pairwise multiple comparisons to determine significant differences (*p* < 0.05). Data bars with different letters are significantly different (*p* < 0.05); data with the same letters are not significantly different (*p* > 0.05).

## 3. Results

### 3.1. Choice of Fluorescent Substrate

**Figure 1:**
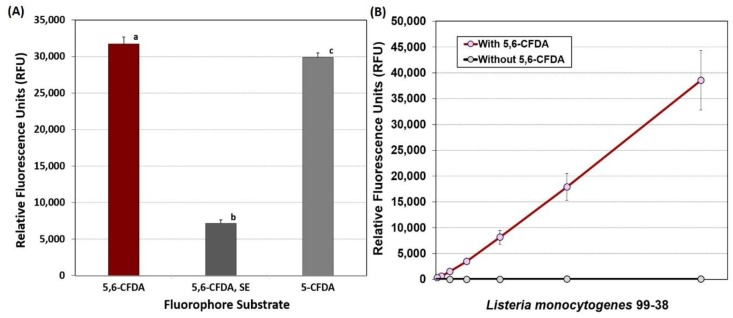
(**A**) Comparison of fluorescence signals obtained using *L. monocytogenes* 99-38 in microplate fluorescence assay with 5,6-CFDA, 5,6-CFDA, SE, or 5-CFDA. Data are presented as the mean of triplicate replications and error bars represent the standard deviation from the mean. Means with different letters are significantly different as determined by one-way ANOVA using the Holm-Sidak test for pairwise multiple comparisons to determine significant differences (*p* < 0.05). (**B**) Two-fold dilutions of planktonic cells incubated with 5,6-CFDA compared to cells without 5,6-CFDA and examined for fluorescence signals (Ex/Em: 485/535 nm). Error bars are the standard deviation of the means of triplicate replications.

We obtained significantly lower levels of fluorescence using 5,6-CFDA, SE and 5-CFDA compared to fluorescence obtained with 5,6-CFDA (Figure 1A). The 5,6-CFDA fluorophore was also examined for whether the level used was limiting when using high levels of bacterial cells as observed during enzymatic detachment. When 2-fold dilutions of planktonic bacteria (~9-log CFU/mL) were incubated with the same amount of 5,6-CFDA as used in adherence assays, no loss of signal linearity was observed (Figure 1B).

### 3.2. Screening of Listeria monocytogenes, Salmonella, and E. coli O157:H7 via Fluorescence Microplate Assay

**Figure 2:**
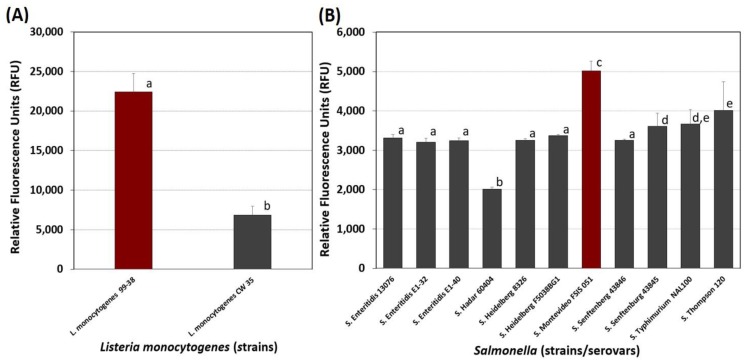
Comparison of adherence of (**A**) *Listeria monocytogenes* and (**B**) *Salmonella* by microplate fluorescence assay with 5,6-CFDA. Data are presented as the mean of triplicate replications and error bars represent the standard deviation from the mean. Means with different letters are significantly different as determined by one-way ANOVA using the Holm-Sidak test for pairwise multiple comparisons to determine significant differences (*p* < 0.05); means with the same letter are not significantly different (*p* > 0.05).

The adherence characteristics of numerous strains of *L. monocytogenes* isolated from ready-to-eat meat processing facilities had previously been examined [27]. In this study, we again confirmed that *L. monocytogenes* 99-38 was a strongly-adherent strain in comparison with weakly-adherent *L. monocytogenes* CW35 (Figure 2A).

Fluorescence microplate assays of various serovars of *Salmonella* from our culture collection demonstrated that *Salmonella* Montevideo FSIS 051 was the most adherent strain and could be useful in development of microplate *Salmonella*-based biofilms to be challenged with various sanitizers in a convenient microplate format (Figure 2B).

**Figure 3:**
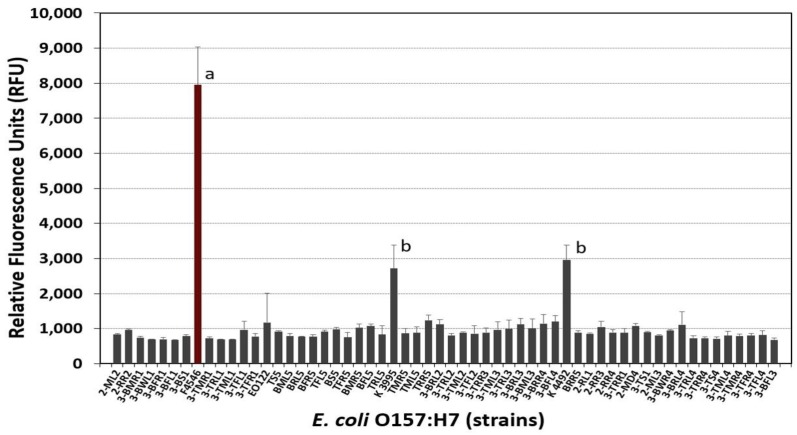
Comparison of adherence of various strains of *E. coli* O157:H7 by microplate fluorescence assay with 5,6-CFDA. Data are presented as the mean of triplicate replications and error bars represent the standard deviation from the mean. Means with different letters are significantly different as determined by one-way ANOVA using the Holm-Sidak test for pairwise multiple comparisons to determine significant differences (*p* < 0.05); means with the same letter are not significantly different (*p* > 0.05).

We also examined a variety of *E. coli* O157:H7 strains isolated from cattle trucks and a known biofilm forming *E. coli* O157:H7 strain (F4546) to screen for strong adherence using the fluorescent adherence assay (Figure 3). Although some strains were moderately adherent (K-3995, K-4492), none were as strongly adherent as the *E. coli* O157:H7 F4546 control strain that is known for adherence (Figure 3)

### 3.3. Buffer Washes with Microplate Biofilms

**Figure 4:**
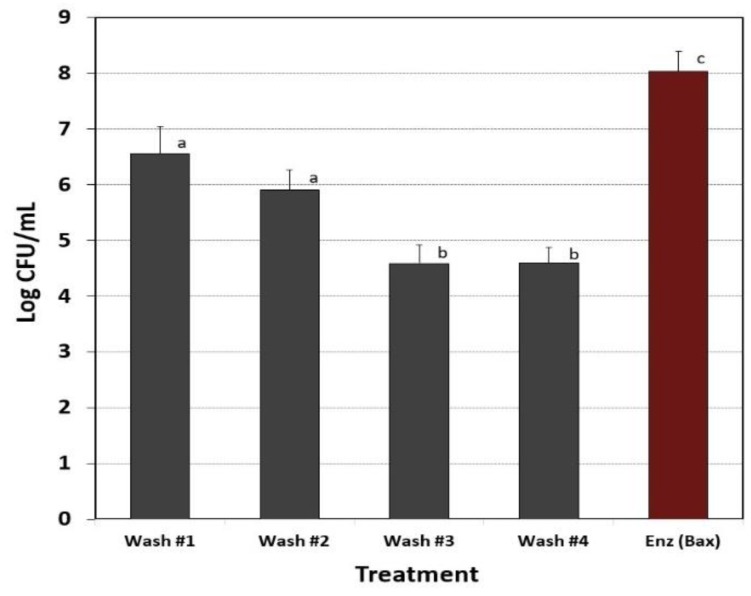
Enumeration of viable cells after multiple buffer washes with 0.05 M Tris buffer (pH 7.4) and after Bax protease treatment (after final wash) of *L. monocytogenes* 99-38 microplate biofilms. Data are presented as the mean of triplicate replications and error bars represent the standard deviation from the mean. Means with different letters are significantly different as determined by one-way ANOVA using the Holm-Sidak test for pairwise multiple comparisons to determine significant differences (*p* < 0.05); means with the same letter are not significantly different (*p* > 0.05).

Biofilms of *L. monocytogenes* were examined for a number of washes required to remove ‘loosely retained’ cells prior to enzymatic treatment for biofilm enumeration. When subjected to 4 rounds of buffer washes using an automated ‘shaking step’, we found that 3 washes were sufficient to remove loose cells and further washes did not further diminish the levels of cells that are leaching from the biofilm (Figure 4).

**Figure 5:**
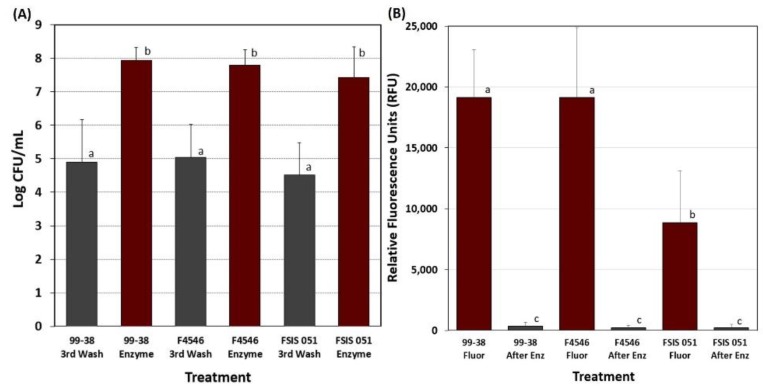
Comparison of enumeration and fluorescence data with *L. monocytogenes* 99-38, *Salmonella* Montevideo FSIS 051, and *E. coli* F4546, before and after enzyme treatment of microplate biofilms. (**A**) Cell enumeration after 3^rd^ round wash buffer followed by Bax protease release of adhered cells from microplates. (**B**) Fluorescence of biofilms with 5,6-CFDA before and after Bax protease treatment to release bacterial cells. Data are presented as the mean of quadruple replications and error bars represent the standard deviation from the mean. Significant differences are between treatments with the same strain. Means with different letters are significantly different as determined by one-way ANOVA using the Holm-Sidak test for pairwise multiple comparisons to determine significant differences (*p* < 0.05).

A side-by-side comparison was also made of the 3 pathogens selected from the prior screening efforts: *L. monocytogenes* 99-38, *E. coli* O157:H7 F4546, and *S.* Montevideo FSIS 051 using the same wash procedure and Bax protease enumeration method (Figure 5A) while using the fluorescent substrate assay to examine levels before and after recovery from microplates (Figure 5B).

### 3.4. Development of Extended Biofilms in Microplates

**Figure 6:**
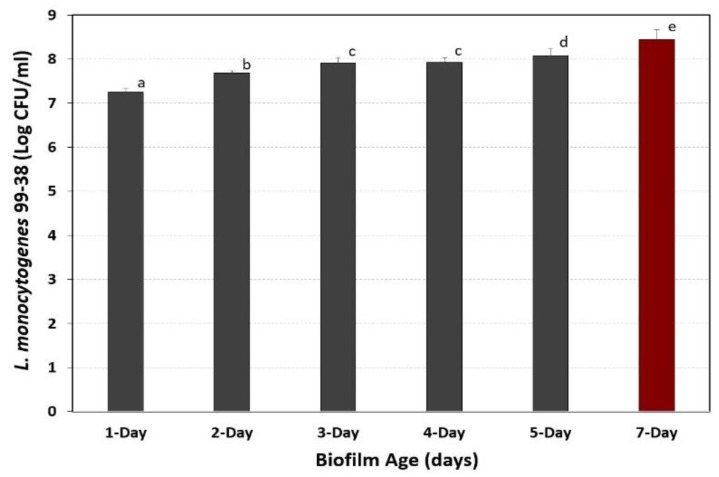
Enumeration of *L. monocytogenes* 99-38 biofilm levels over time after repeated incubation in microplates. Planktonic cells were removed daily, washed with buffer, and replaced with fresh sterile media. Attached cells were enumerated by detachment with pronase E and represented as the means of triplicate replications; error bars represent the standard deviation of the means. Means with different letters are significantly different as determined by one-way ANOVA using the Holm-Sidak test for pairwise multiple comparisons to determine significant differences (*p* < 0.05); means with the same letter are not significantly different (*p* > 0.05).

In previous studies, a short 2–3 day cycle of repeated microplate washing/incubation was used to establish microplate biofilms [19]. In this study, conditions were sought that would achieve a robust 7-day biofilm for use in upcoming studies to examine the effect of sanitizers on biofilms of foodborne pathogens. This was done by repeated cycles of growth, adherence, washing away of planktonic/loose cells, and addition of sterile media to continue growth from those cells attached to microplate surfaces. In prior studies, several enzymes were used to recover and enumerate attached bacterial cells from microplates such as pronase E (Figure 6) and Bax protease [19]. The data shows that we were able to incrementally increase the level of adhered cells by >12-fold over 7-days using the ‘extended biofilm’ approach to create a more robust and challenging biofilm (Figure 6).

### 3.5. Evaluation of Various Enzymes for Bacterial Detachment and Enumeration

**Figure 7:**
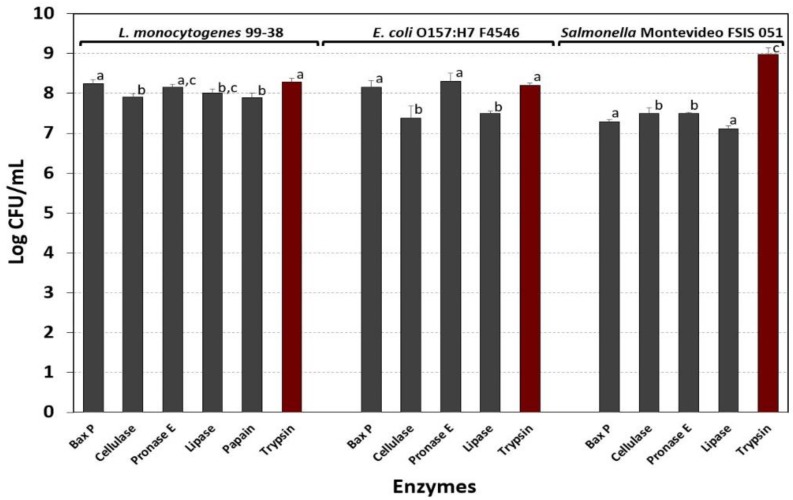
Recovery and enumeration of *L. monocytogenes* 99-38, *E. coli* O157:H7 F4546, and *Salmonella* Montevideo FSIS 051 biofilms after treatment with various enzymes (Bax protease, cellulase, pronase E, papain, trypsin, or lipase). Data bars represent the means of triplicate replications and error bars represent standard deviation of the means. Means with different letters are significantly different as determined by one-way ANOVA using the Holm-Sidak test for pairwise multiple comparisons within the same organism to determine significant differences (*p* < 0.05); means with the same letter are not significantly different (*p* > 0.05).

In prior assays, both Bax protease [19] (Figure 4 and Figure 5) and pronase E (Figure 6) were used for detaching cells from biofilms for enumeration. Although Bax protease was effective, the identity of the particular protease in this proprietary commercial reagent was unknown. It was also important to re-evaluate which enzyme(s) worked best to enumerate cell levels from microplate assays with *L. monocytogenes*, *S.* Montevideo, and *E. coli* O157:H7 biofilms in upcoming studies. Although all the enzymes tested worked sufficiently well to obtain high level cell counts from 7-day biofilms, three were nearly equal for both *L. monocytogenes* 99-38 and *E. coli* O157:H7 F4546, including Bax protease, pronase E, and trypsin (Figure 7). However, for *Salmonella*, trypsin was significantly better than the other enzymes (Figure 7).

## 4. Discussion

Biofilms have been measured in various ways, including absorbance measurements taken directly with a plate reader from biofilms in microplates stained with crystal violet [31,32], or indirectly from dye recovered by de-staining solutions [33,34], after vortexing with glass beads to recover cells for enumeration [35] or after enzyme treatment and sonication to dislodge cells [36]. Microplate adherence and fluorescence visualization was examined to mimic flow cytometry whereby individual cells are ‘counted’ based on fluorescence of internalized fluorophores as they pass through a capillary tube that is transected by a laser. In our work, this method was modified for assessment of adherent cells attached in situ to the walls of microplate wells using a fluorescence plate reader as a qualitative and quick screening procedure.

In prior and current studies, ‘non-treated’ microplates were used to assess the bacterial strains’ inherent and unaided ability to adhere (i.e., ‘treated’ plates are often used with tissue culture assays to promote adherence). It is likely that treated plates could subsequently be used to provide even more formidable biofilms for use in challenge studies with antimicrobials and sanitizers. Flat-bottom microplates are also important when using microplate washers as those with curved bottoms might interfere with lowering of the paired needles of the plate washer into the plates and/or leave significant amounts of wash fluid behind.

Fluorescein is a fluorescing compound that can freely diffuse through cell membranes. Its use became popularized by applications in flow cytometry [37], vital staining of live/dead bacterial cells [38], and applications allowing visualization by microscopy and studies in apoptosis [39]. Unmodified fluorescein can fluoresce externally when applied to bacterial cells whereas the signal can be quenched by modification with diacetate (i.e., 5,6-CFDA). Once internalized, cytoplasmic esterases hydrolyze the fluorophore, causing a significant spike in intracellular fluorescence. The purported benefit of the succinyl ester of 5,6-CFDA (5,6-CFDA, SE) was that in addition to diacetate, the -SE modification allows it to be retained longer intracellularly because of the propensity to bind to amino groups. Fuller et al. [40] used 5,6-CFDA,SE to follow the fate of labelled bacterial cells under no growth conditions for 28 days in groundwater sediment microcosms as they retained fluorescence during this time period. We contemplated whether this feature would allow us to achieve higher and more sensitive detection levels. However, 5,6-CFDA not only performed better than 5-CFDA or 5,6-CFDA, SE (Figure 1A), but was also significantly less expensive than the other fluorophores. The use of 5,6-CFDA in our application has been to qualitatively indicate the degree of adherence of cells as in a biofilm when screening numerous strains. When 5,6-CFDA was mixed with serial dilutions of planktonic cells, we observed a linearity of signal even with the least diluted (highest) level of cells suggesting that the levels used in our biofilm assays were not limiting (Figure 1B). The fact that biofilm-adhered cells are possibly diffusion limited compared to planktonic cells further suggests that levels of fluorophore are not limiting in our biofilm assays.

In establishing conditions for *Listeria, Salmonella,* and *E. coli* biofilms, *L. monocytogenes* 99-38 was confirmed [19] as a robust, strongly-adherent strain that would serve well in the formation of biofilms for challenge studies (Figure 2A). Strains of *E. coli* O157:H7 and *Salmonella* in our collection were further screened in search of good biofilm formers in preparation of a project to evaluate the effect of sanitizers on biofilms of *L. monocytogenes, Salmonella* spp.*,* and *E. coli* O157:H7. Among the *Salmonella* serovars in our collection, *Salmonella* Montevideo FSIS 051 was significantly more adherent than the other strains tested (Figure 2B). *E. coli* F4546, a strain implicated in biofilm formation [41], was also the most adherent from among the 58 strains of *E. coli* O157:H7 tested (Figure 3). Based on the fluorescent microplate data with these strains, we continued optimization of biofilm adherence with *L. monocytogenes* 99-38, *Salmonella* Montevideo FSIS 051, and *E. coli* F4546 (Figure 2 and Figure 3).

Microplate biofilms comprised of *L. monocytogenes* 99-38 were washed multiple times with buffer before enzymatic treatment to remove the bacterial culture media, planktonic, and loosely adhered cells. The plate washer had a built-in ‘shake’ mode that provided a standardized shaking regimen to release loosely held cells, but it is possible that this process will always free up cells. It was decided that as the level of cells stabilized (i.e., after 2–4 washes), that this was indicative of a sufficient degree of washing (Figure 4). The chosen wash parameter was examined for all 3 of the selected strains (99-39, FSIS 051, and F4546) to insure that loosely held cells were removed (Figure 5A). Although high levels of cells were still recovered in the wash buffer, they represented a small proportion of the levels attached to the well surfaces (Figure 5A). Similar ‘before and after’ trials using fluorescence assays were performed with a full biofilm load and were compared to similar wells after detachment of cells with protease, washing with buffer, and application of fluorescent substrate (Figure 5B). The absence of significant detectable fluorescent signal after enzyme treatment supports the data obtained with enumeration after enzymatic detachment. The application of 5,6-CFDA does not affect the viability of cells, and therefore wells treated with 5,6-CFDA could be used directly after fluorescence measurements for enzyme treatment and microbial platings.

*Bacillus* isolates from a dairy processing facility were recently shown to produce thick biofilms that were resistant to routine clean-in-place procedures [42]. Similarly, our intention was to increase the robustness of our biofilms so that they would be a better challenge in upcoming studies evaluating commercial sanitizers as is being done by others [43]. We did this by selecting strains that were superior in their initial adhesion to surfaces, and then used a daily cycling of growth in media, buffer wash, fresh media, and further incubation that was repeated for 7 days to provide a more robust biofilm. Microplate biofilms were examined in 1-to-7-day increments (i.e., different plates for the respective days) by enzymatic detachment and enumeration of cell levels to insure that incremental increases were occurring during the extended cycle times (Figure 6). Since enzymatic recovery of biofilms from *E. coli* or *Salmonella* were not examined previously, we re-examined the application of hydrolytic enzymes for all 3 organisms in microplate biofilm assays. Bax protease, pronase E, and trypsin enumerated comparable levels of detached cells for *L. monocytogenes* 99-38 and *E. coli* F4546 (Figure 7). However, trypsin was significantly better in providing higher counts than the other enzymes with *Salmonella* Montevideo FSIS 051 and was the enzyme of choice going forward (Figure 7). Trypsin has long been used in tissue culture studies for releasing tissue culture cells from flasks [44], and it is the least expensive of the enzymes used in this study and is sold in a convenient liquid form. It is becoming more evident that biofilms are among the root causes of many recurring foodborne illness outbreaks and spoilage contaminations [45], and enzymes are being further exploited to address them [46]. The complex heterogeneity in the composition of some biofilms may ultimately require unique biological enzymes [47,48] or combinations of enzyme and chemical treatment [49] to eradicate troublesome biofilms.

## 5. Conclusions

This improved microplate biofilm assay will be useful for enumerating initial and residual viable cells in studies on biofilms of *L. monocytogenes, Salmonella*, and/or *E. coli* O157 that currently present recurring problems in food processing facilities. Trypsin and 5,6-CFDA are both the most effective and least expensive of the alternative components we examined. Bacteria continue to leach from such biofilms, and mimics similar situations in food processing facilities where potential biofilms on food contact surfaces may contaminate passing foods as foci of contamination by the sloughing off of loosely-held cells. We hope to examine the effect of commercial sanitizers using these challenge organisms to identify those that might be more effective against biofilms.

## References

[1] Zhao X., Zhao F., Wang J., Zhong N. (2017). Biofilm formation and control strategies of foodborne pathogens: Food safety perspectives. RSC Adv..

[2] Achinas S.C.N., Euverink G.J.W. (2019). A brief recap of microbial adhesion and biofilms. Appl. Sci..

[3] Stoodley P., Sauer K., Davies D.G., Costerton J.W. (2002). Biofilms as complex differentiated communities. Annu. Rev. Microbiol..

[4] Hinsa S.M., Espinosa-Urgel M., Ramos J.L., O’Toole G.A. (2003). Transition from reversible to irreversible attachment during biofilm formation by *Pseudomonas fluorescens* WCS365 requires an ABC transporter and a large secreted protein. Mol. Microbiol..

[5] Wagner S., Hauck D., Hoffmann M., Sommer R., Joachim I., Müller R., Imberty A., Varrot A., Titz A. (2017). Covalent lectin inhibition and application in bacterial biofilm imaging. Angew. Chem. Int. Ed..

[6] Beloin C., Roux A., Ghigo J.M. (2008). *Escherichia coli* biofilms. Curr. Top. Microbiol. Immunol..

[7] Kumar C.G., Anand S.K. (1998). Significance of microbial biofilms in food industry: A review. Int. J. Food Microbiol..

[8] Davies D.G., Parsek M.R., Pearson J.P., Iglewski B.H., Costerton J.W., Greenberg E.P. (1998). The Involvement of cell-to-cell signals in the development of a bacterial biofilm. Science.

[9] Cao S., Wang J., Chen H., Chen D. (2011). Progress of marine biofouling and antifouling technologies. Chin. Sci. Bull..

[10] Hallam N.B., West J.R., Forster C.F., Simms J. (2001). The potential for biofilm growth in water distribution systems. Water Res..

[11] Hall-Stoodley L., Costerton J.W., Stoodley P. (2004). Bacterial biofilms: From the natural environment to infectious diseases. Nat. Rev. Microbiol..

[12] Marsh P.D. (2006). Dental plaque as a biofilm and a microbial community—implications for health and disease. BMC Oral Health.

[13] Chmielewski R.A.N., Frank J.F. (2003). Biofilm formation and control in food processing facilities. Compr. Rev. Food Sci. Food Saf..

[14] Lee Wong A.C. (1998). Biofilms in food processing environments. J. Dairy Sci..

[15] Alvarez-Ordóñez A., Coughlan L.M., Briandet R., Cotter P.D. (2019). Biofilms in food processing environments: Challenges and opportunities. Annu. Rev. Food Sci. Technol..

[16] O’Toole G.A., Kolter R. (1998). Initiation of biofilm formation in *Pseudomonas fluorescens* WCS365 proceeds via multiple, convergent signalling pathways: A genetic analysis. Mol. Microbiol..

[17] Merritt J.H., Kadouri D.E., O’Toole G.A. (2005). Growing and analyzing static biofilms. Curr. Protoc. Microbiol..

[18] Hartmann R., Singh P.K., Pearce P., Mok R., Song B., Díaz-Pascual F., Dunkel J., Drescher K. (2019). Emergence of three-dimensional order and structure in growing biofilms. Nat. Phys..

[19] Gamble R., Muriana P.M. (2007). Microplate fluorescence assay for measurement of the ability of strains of *Listeria monocytogenes* from meat and meat-processing plants to adhere to abiotic surfaces. Appl. Environ. Microbiol..

[20] Hood S.K., Zottola E.A. (1995). Biofilms in food processing. Food Control.

[21] Kaplan J.B. (2010). Biofilm dispersal: Mechanisms, clinical implications, and potential therapeutic uses. J. Dent. Res..

[22] Selan L., Berlutti F., Passariello C., Comodi-Ballanti M.R., Thaller M.C. (1993). Proteolytic enzymes: A new treatment strategy for prosthetic infections?. Antimicrob. Agents Chemother..

[23] Zhang G., Ma L., Beuchat L.R., Erickson M.C., Phelan V.H., Doyle M.P. (2009). Evaluation of treatments for elimination of foodborne pathogens on the surface of leaves and roots of lettuce (*Lactuca sativa* L.). J. Food Prot..

[24] Cui Y., Liu D., Chen J. (2018). Fate of various *Salmonella enterica* and enterohemorrhagic *Escherichia coli* cells attached to alfalfa, fenugreek, lettuce, and tomato seeds during germination. Food Control.

[25] Juneja V.K., Valenzuela Melendres M., Huang L., Gumudavelli V., Subbiah J., Thippareddi H. (2007). Modeling the effect of temperature on growth of *Salmonella* in chicken. Food Microbiol..

[26] Tiong H., Hartson S., Muriana P.M. (2016). Comparison of surface proteomes of adherence variants of *Listeria monocytogenes* using LC-MS/MS for identification of potential surface adhesins. Pathogens.

[27] Kushwaha K., Muriana P.M. (2009). Adherence Characteristics of *Listeria* Strains Isolated from Three Ready-to-Eat Meat Processing Plants. J. Food Prot..

[28] Juneja V.K., Eblen B.S., Ransom G.M. (2001). Thermal inactivation of *Salmonella* spp. in chicken broth, beef, pork, turkey, and chicken: Determination of D- and Z-values. J. Food Sci..

[29] Alonso E.P.C., Gilliland S.E., Krehbiel C.R. (2007). Incidence and toxin production ability of *Escherichia coli* O157:H7 isolated from cattle trucks. J. Food Prot..

[30] Pericolini E., Colombari B., Ferretti G., Iseppi R., Ardizzoni A., Girardis M., Sala A., Peppoloni S., Blasi E. (2018). Real-time monitoring of *Pseudomonas aeruginosa* biofilm formation on endotracheal tubes in vitro. BMC Microbiol..

[31] Stepanović S., Ćirković I., Ranin L., Svabić-Vlahović M. (2004). Biofilm formation by *Salmonella* spp. and *Listeria monocytogenes* on plastic surface. Lett. Appl. Microbiol..

[32] Stepanovic S., Vukovic D., Hola V., Di Bonaventura G., Djukic S., Cirkovic I., Ruzicka F. (2007). Quantification of biofilm in microtiter plates: Overview of testing conditions and practical recommendations for assessment of biofilm production by staphylococci. Acta Pathol. Microbiol. Immunol. Scand..

[33] Djordjevic D., Wiedmann M., McLandsborough L.A. (2002). Microtiter plate assay for assessment of *Listeria monocytogenes* biofilm formation. Appl. Environ. Microbiol..

[34] Borucki M.K., Peppin J.D., White D., Loge F., Call D.R. (2003). Variation in biofilm formation among strains of *Listeria monocytogenes*. Appl. Environ. Microbiol..

[35] Giaouris E., Chorianaopoulos N., Nychas G.-J.E. (2005). Effect of temperature, pH, and water activity on biofilm formation by *Salmonella enterica* Enteritidis PT4 on stainless steel surfaces as indicated by the bead vortexing method and conductance measurements. J. Food Prot..

[36] Oulahal-Lagsir N., Martial-Gros A., Bonneau M., Blum L.J. (2003). “*Escherichia coli*-milk” biofilm removal from stainless steel surfaces: Synergism between ultrasonic waves and enzymes. Biofouling.

[37] Breeuwer P., Drocourt J.L., Bunschoten N., Zwietering M.H., Rombouts F.M., Abee T. (1995). Characterization of uptake and hydrolysis of fluorescein diacetate and carboxyfluorescein diacetate by intracellular esterases in *Saccharomyces cerevisiae*, which result in accumulation of fluorescent product. Appl. Environ. Microbiol..

[38] Bunthof C.J., Bloemen K., Breeuwer P., Rombouts F.M., Abee T. (2001). Flow cytometric assessment of viability of lactic acid bacteria. Appl. Environ. Microbiol..

[39] Farinacci M. (2007). Improved apoptosis detection in ovine neutrophils by annexin V and carboxyfluorescein diacetate staining. Cytotechnology.

[40] Fuller M.E., Streger S.H., Rothmel R.K., Mailloux B.J., Hall J.A., Onstott T.C., Fredrickson J.K., Balkwill D.L., DeFlaun M.F. (2000). Development of a vital fluorescent staining method for monitoring bacterial transport in subsurface environments. Appl. Environ. Microbiol..

[41] Perez-Conesa D., McLandsborough L., Weiss J. (2006). Inhibition and inactivation of *Listeria monocytogenes* and *Escherichia coli* O157:H7 colony biofilms by micellar-encapsulated eugenol and carvacrol. J. Food Prot..

[42] Ostrov I.P.T., Shemesh M. (2019). Robust biofilm-forming *Bacillus* isolates from the dairy environment demonstrate an enhanced resistance to cleaning-in-place procedures. Foods.

[43] Rajamani S., Sandy R., Kota K., Lundh L., Gomba G., Recabo K., Duplantier A., Panchal R.G. (2019). Robust biofilm assay for quantification and high throughput screening applications. J. Microbiol. Methods.

[44] Phelan K., May K.M. (2015). Basic techniques in mammalian cell tissue culture. Curr. Protoc. Cell Biol..

[45] Galié S., García-Gutiérrez C., Miguélez E.M., Villar C.J., Lombó F. (2018). Biofilms in the food industry: Health aspects and control methods. Front. Microbiol..

[46] Nahar S., Mizan M.F.R., Ha A.J.-W., Ha S.-D. (2018). Advances and future prospects of enzyme-based biofilm prevention approaches in the food industry. Compr. Rev. Food Sci. Food Saf..

[47] Daboor S.M., Raudonis R., Cohen A., Rohde J.R., Cheng Z. (2019). Marine bacteria, a source for alginolytic enzyme to disrupt *Pseudomonas aeruginosa* biofilms. Mar. Drugs.

[48] Saggu S.K., Jha G., Mishra P.C. (2019). Enzymatic degradation of biofilm by metalloprotease from *Microbacterium* sp. SKS10. Front. Bioeng. Biotechnol..

[49] Lim E.S., Koo O.K., Kim M.-J., Kim J.-S. (2019). Bio-enzymes for inhibition and elimination of *Escherichia coli* O157:H7 biofilm and their synergistic effect with sodium hypochlorite. Sci. Rep..

